# Fano-Resonant Metasurface with 92% Reflectivity Based on Lithium Niobate on Insulator

**DOI:** 10.3390/nano12213849

**Published:** 2022-10-31

**Authors:** Leshu Liu, Ken Liu, Ning Liu, Zhihong Zhu, Jianfa Zhang

**Affiliations:** College of Advanced Interdisciplinary Studies & Hunan Provincial Key Laboratory of Novel Nano-Optoelectronic Information Materials and Devices, National University of Defense Technology, Changsha 410073, China

**Keywords:** lithium niobate on insulator, metasurface, high reflectivity, Fano-resonant, Fabry–Perot cavity

## Abstract

Lithium niobate is an excellent optoelectronic and nonlinear material, which plays an important role in integrated optics. However, lithium niobate is difficult to etch due to its very stable chemical nature, and the microstructure of lithium niobate’s metasurface is generally of subwavelength, which further increases its processing difficulty. Here, by using Ar^+^-based inductively coupled plasma etching and KOH wet etching, we improve the etching quality and fabricate a Fano-resonant metasurface based on lithium niobate on insulator, which has a very high reflectivity of 92% at near-infrared wavelength and the potential of becoming a high-reflectivity film. In addition, to evaluate the practical performance of the metasurface, we constructed a Fabry–Perot cavity by using it as a cavity mirror, whose reflection spectrum shows a finesse of 38. Our work paves the way for the development of functional metasurfaces and other advanced photonic devices based on lithium niobate on insulator.

## 1. Introduction

Lithium niobate (LiNbO_3_, LN) has become one of the most attractive platforms in integrated optics over the last few decades due to its excellent optoelectronic and material properties, such as a wide transparent window (0.4 μm–5 μm), strong electro-optical effects and nonlinear effects, and so on [1,2,3]. Traditionally, most LN waveguide devices are prepared based on ion diffusion or proton exchange processes, which are extremely poor at limiting light (Δn < 0.02). As a result, these devices are usually bulky and high-loss, severely limiting the development of LN integrated photonics. Fortunately, with the rapid development of micro/nano processing technology and the emergence of lithium niobate on insulator (LNOI) [4], researchers have proposed various processing techniques for thin-film LN, such as thermal diffusion or plasmon exchange [5], femtosecond laser direct writing [6,7], focused ion beam etching [8], and plasma dry etching [9]. Thanks to these advanced processing techniques, a variety of high-performance integrated photonic devices based on LNOI have been proposed, including low-loss waveguides [10], electro-optical modulators [11,12], micro-resonators [13,14], metamaterials [15], nonlinear optical devices [16,17], and so on. These research findings have greatly contributed to the prosperity and development of integrated photonics based on LNOI. However, LN is difficult to etch due to its extremely stable chemical properties, and the microstructure of an LN metasurface is generally of subwavelength, which further increases its processing difficulty.

Metamaterials are artificial structures consisting of a series of subwavelength meta-atoms. In contrast to natural crystals, their ‘meta-atoms’ and the arrangement between them can be freely designed to obtain arbitrary electromagnetic responses [18], such as negative refraction [19], zero refraction [20], etc. Metasurfaces, as a two-dimensional form of metamaterials, not only have the excellent properties of metamaterials but also have the advantages of a monolayer and simple processing, and thus they are attracting wide attention [21]. Initially, metasurfaces were made of metallic materials [22,23]. The free electrons in metals oscillate collectively under the influence of external electromagnetic waves, resulting in resonances that greatly enhance the interaction between light and matter [24]. However, the inherent ohmic loss of metallic materials and the inability of plasmon resonance to operate at high frequencies hindered the application and development of metallic metasurfaces. Later, all-dielectric metasurfaces gradually became trendy because a dielectric has almost no absorption in visible and near-infrared ranges, and more importantly, the electromagnetic properties of all-dielectric metasurfaces usually come from the Mie scattering of subwavelength resonant structures, which are not limited by the plasma resonance frequency [25,26]. Many novel nanophotonic devices based on all-dielectric metasurfaces have been reported in recent years, such as metalenses [27], invisible cloaks [28,29], beam splitters [30,31], and Huygens surfaces [32,33], which have provided a large amount of knowledge regarding the development of LN metasurfaces.

**Figure 1:**
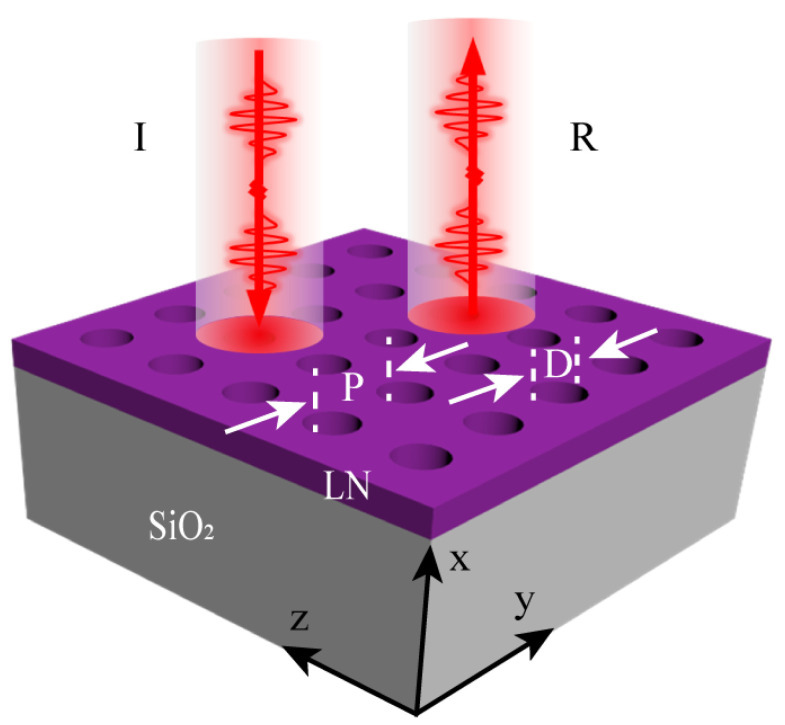
Schematic of the high-reflectivity metasurface based on x-cut LNOI comprising a square lattice of air holes, where P and D denote the period of the metasurface and the diameter of the air hole, respectively.

In this paper, we report the fabrication of a Fano-resonance metasurface with a high reflectivity based on LNOI by using Argon-based (Ar^+^-based) inductively coupled plasma (ICP) etching and KOH wet etching. As shown in Figure 1, this metasurface consists of a series of periodically arranged air holes with a period (P) and diameter (d) of 1050 nm and 525 nm, respectively. When a y-polarized plane wave is incident perpendicular to the LN metasurface, the peak reflectivity is 92%. To better evaluate the practical performance of the metasurface, we constructed a Fabry–Perot cavity (F-P cavity) with the LN metasurface and another dielectric mirror with a reflectivity close to 1. The finesse of the reflectance spectrum of the F-P cavity is 38, which shows a very high application potential. Our work paves the way for the development and application of functional metasurfaces and other advanced photonic devices based on LNOI.

## 2. Materials and Methods

LN is a uniaxial crystal, and the choice of its optical axis orientation affects the performance of the device. In this work, we chose a 300 nm thick commercial x-cut LNOI wafer (from NANOLN), where the thicknesses of the silica and silicon layers were 4 μm and 500 μm, respectively.

**Figure 2:**
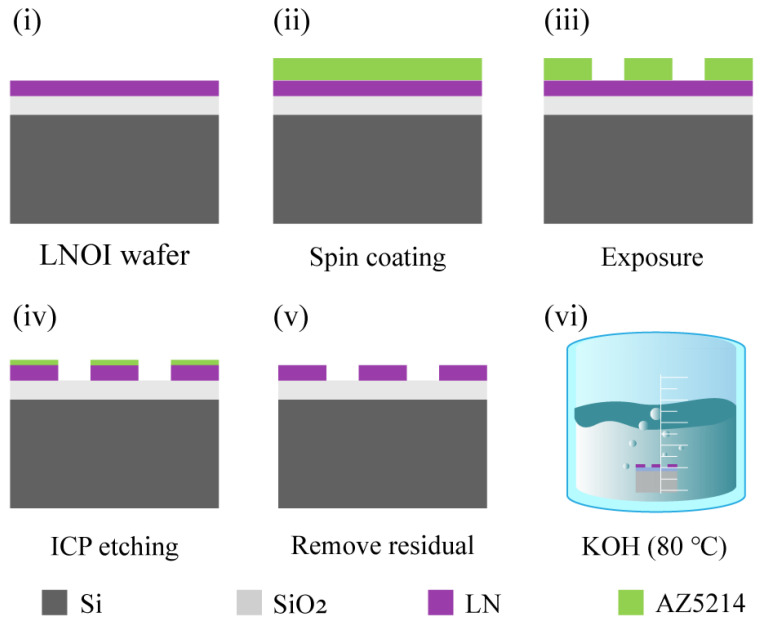
The device fabrication process. (**i**) The x-cut LNOI wafer; (**ii**): Spin-coating photoresist; (**iii**) Defining the pattern of the LN metasurface by Stepper lithography; (**iv**) Transferring the pattern of the LN metasurface onto the LN layer by inductively coupled plasma (ICP) etching; (**v**) Removing the residual resist; (**vi**) The fabricated LN metasurface was immersed in 0.5 mol/L KOH solution at 80 °C for 20 min to further improve the etching quality.

Figure 2 shows the detailed fabrication process of the LN metasurface. First, the LNOI wafers are split to obtain a single small chip with a size of 5 × 5 mm^2^ and cleaned sequentially with acetone, isopropanol, and deionized water. After these developments, a layer of 1.4 μm photoresist (AZ5214) is firstly spin-coated on top of the LNOI (spin-coating speed is 4000 rpm). Next, the LN metasurface is patterned on the photoresist layer by Stepper lithography (the exposure time and light intensity are 245 ms and 600 mW/cm^2^, respectively). Then, we used Ar^+^-based ICP etching to transfer the pattern onto the LN layer. The specific etching parameters are as follows: RF power (200 W)/RF bias (100 W)/chamber pressure (5 mTorr)/Ar gas stream (40 sccm). In this condition, the selectivity and etching rate are about 1:1 and 20 nm/min, respectively. Finally, the residual photoresist is removed by using O_2_ plasma (300 W/2 min) to obtain the LN metasurface.

**Figure 3:**
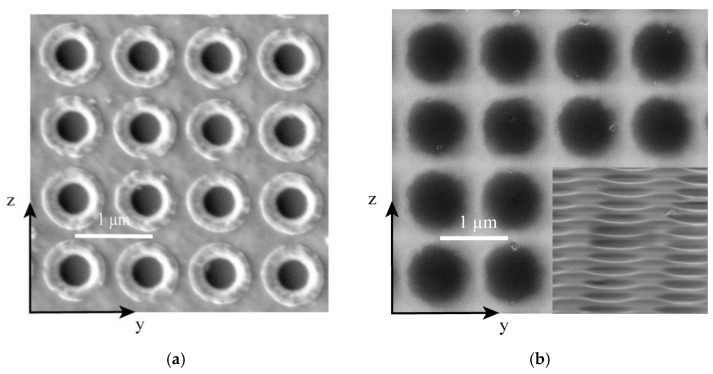
Scanning electron micrograph (SEM) images of the fabricated LN metasurfaces along the yz plane. (**a**) The top-view SEM image of the fabricated LN metasurface after ICP etching and before KOH wet etching; (**b**) The top-view SEM image of the fabricated LN metasurface after KOH wet etching; the inset is the tilt-10° view image.

The fabricated LN metasurface is shown in Figure 3a. From the SEM image, we can see that there are many redeposits around the air holes, and the surface is rough, which will aggravate the light-scattering effect and thus seriously affect the performance of the LN metasurface. To further enhance the smoothness of the surface and the performance of the LN metasurface, we soaked it in a 0.5 mol/L KOH solution at 80 °C for 20 min. Figure 3b shows the top-view and tilt-10° view (inset) SEM images of the metasurface after KOH wet etching, which shows that the redeposits around the air holes are almost removed and the etched surface of the LN metasurface is significantly improved.

## 3. Results

### 3.1. LN Metasurface

**Figure 4:**
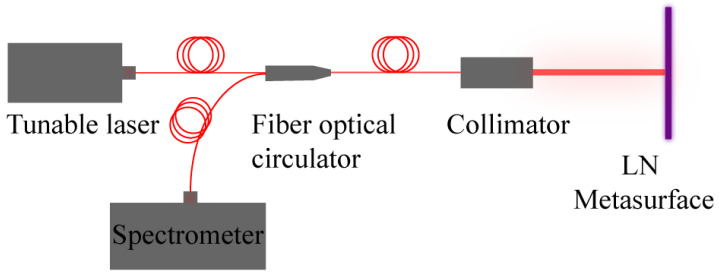
Reflectivity measurement optical path of LN metasurface.

In order to characterize the performance of the LN metasurface, we built a measurement optical path as shown in Figure 4.

**Figure 5:**
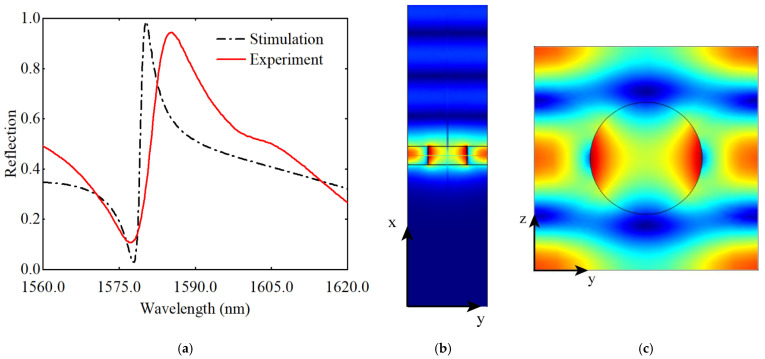
(**a**) The reflection of the LN metasurface in the wavelength range of 1560–1620 nm. The red line and black line are the experimental and simulated results, respectively; (**b**) Optical field distribution in xy plane; (**c**) Optical field distribution in yz plane.

In this optical path, we use a tunable laser as the light source, and its output power and tuning range are set to 1 mW and 1560–1620 nm, respectively. When the system starts to operate, the laser beam is firstly coupled into a fiber-optical circulator through a single-mode fiber. The fiber-optical circulator is a typical three-port device whose main function is to limit the direction of light transmission. It ensures that the light from the tunable laser is transmitted only forward into the collimator, and the reflected light coming from the metasurface is transmitted only backward to the detector. Thus, when the laser beam from the tunable laser passes through the fiber-optical circulator, it can only be coupled into the collimator through another single-mode fiber and converted into a plane wave. The plane wave will then be incident on the lithium niobate metasurface and reflected. This part of the reflected light will be transmitted backward, passing through the collimator and the fiber circulator in turn before finally becoming incident on the spectrometer by the fiber circulator. In this way, we obtain the reflection power spectrum of the LN metasurface. Then, we pull out the fiber connected between the laser and the fiber-optical circulator and connect it directly to the spectrometer so that the output power spectrum of the laser can be measured. Dividing the above two data sets, we can obtain the reflection of the metasurface in the wavelength range of 1560–1620 nm, and the results are shown in Figure 5a.

The red and black lines indicate the simulated and experimental results in the wavelength range from 1560 nm to 1620 nm, respectively. They exhibit strong Fano resonances near 1580 nm and 1585.2 nm, respectively. The experimentally measured resonance peak reflectance is 92%, which is slightly lower than the simulated results. In addition, there are some differences between the experimental and simulated results in the position and width of the resonance peaks: the width of the experimentally measured resonance peaks is wider than that of the simulated results, and there is a gap of several nm between the experimentally measured resonance peak positions and the simulated results. We speculate that this discrepancy is caused by manufacturing errors. Figure 5b,c shows the optical field distribution in the xy and yz planes at the resonance peak, respectively. The optical field is well confined in the LN layer, and the incident light interferes with the reflected light to form standing waves above the LN layer, which is consistent with the theory.

### 3.2. F-P Cavity

**Figure 6:**
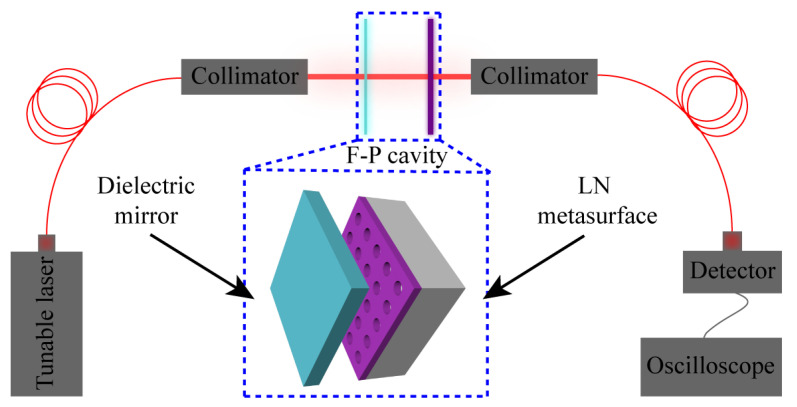
The F-P cavity and its measurement optical path. The F-P cavity is formed by the LN metasurface and another dielectric mirror with reflectivity close to 1.

In addition, to evaluate the practical performance of the metasurface, we constructed an F-P cavity using the LN metasurface and another dielectric mirror with reflectivity close to 1, as shown in the inset in Figure 6.

Figure 6 illustrates the measurement optical path, where the light from the tunable laser is first coupled into a collimator through a single-mode fiber and converted into a plane wave. When these plane waves are incident into the F-P cavity, those that meet the resonance conditions will be reflected back and forth to form standing waves, while those that do not meet the resonance conditions will be directly transmitted through the F-P cavity and then coupled into a single-mode waveguide by another collimator. Finally, the transmitted light will be incident on the detector and recorded by the oscilloscope. In this way, we can obtain the transmission power spectrum of this F-P cavity. It is worth noting that the reflectivity of our designed LN metasurface varies with wavelength, and therefore the peak power varies for each resonant mode of the F-P cavity.

**Figure 7:**
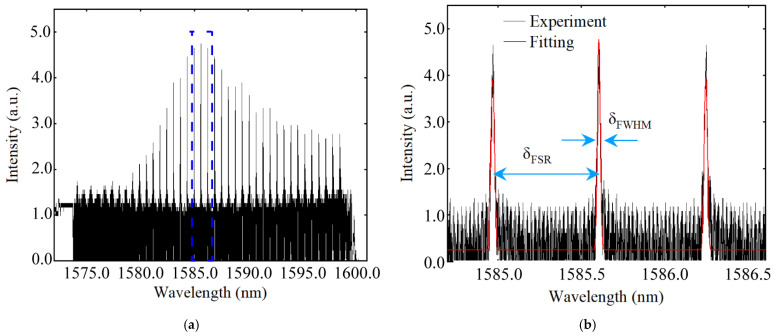
The transmission power spectrum of the F-P cavity. (**a**) The transmission power spectrum of the F-P cavity ranging from 1572–1601 nm when the output power of the tunable laser is 1 mW. The measured FSR is 0.647; (**b**) The partial transmission power spectrum and its fitted curve range from 1584.7 nm to 1586.6 nm. The measured f_R_ is 38 when the measured FWHM is 0.017 near 1585 nm.

Figure 7a shows the transmission power spectrum of the F-P cavity in the wavelength range of 1572–1601 nm. The resonance peak is near 1585 nm and is sharp, which is consistent with our previous measurements. In order to characterize the performance of this F-P cavity, we measured the finesse of its transmission spectrum. Figure 7b shows the transmission power spectrum of the F-P cavity in the wavelength range of 1584.7–1586.6 nm [the part of the blue dashed box in Figure 6]. The black curve is the raw data, and the red curve is the fitted result. The fitted results show that the free spectrum range (FSR) and the full width at half maximum (FWHM) of the F-P cavity are 0.647 and 0.017, respectively. According to the finesse f_R_ of the F-P cavity transmission power spectrum versus the FSR, FWHM and R [34,35],

(1) $$f_{R}=\frac{{\delta \nu }_{\mathrm{FSR}}}{{\delta \nu }_{\mathrm{FWHM}}}=\frac{\pi \sqrt{R}}{1-R}$$

The finesse f_R_ of this F-P cavity is calculated to be 38 based on the transmission power spectrum, which is consistent with the result based on the reflectivity R = 92% of the LN metasurface. This demonstrates the viability of the proposed LN metasurface as an F-P cavity reflector. Moreover, being single-layered and easy to fabricate, it has great potential for many application scenarios that require high-reflectivity dielectric films. 

## 4. Conclusions

In this work, we propose and successfully fabricate a Fano-resonant metasurface based on LNOI by using Ar^+^-based inductively coupled plasma etching and KOH wet etching, which exhibits a high reflectivity of 92% at the resonance peak at 1585.2 nm. In addition, we construct an F-P cavity using the LN metasurface and another dielectric mirror with a reflectivity close to 1. It has a finesse of 38 and shows a strong ability to limit the optical field. At the same time, it has the advantages of being monolayer and easily processed. Thus, it has great potential for various applications. Our work paves the way for the development of functional metasurfaces and other advanced photonic devices based on LNOI.

## Data Availability

The data presented in this study are available on request from the corresponding author.
